# Computational Prediction and Experimental Validation of the Unique Molecular Mode of Action of Scoulerine

**DOI:** 10.3390/molecules27133991

**Published:** 2022-06-21

**Authors:** Mahshad Moshari, Qian Wang, Marek Michalak, Mariusz Klobukowski, Jack Adam Tuszynski

**Affiliations:** 1 Department of Chemistry, University of Alberta, Edmonton, AB T6G 2G2, Canada; moshari@ualberta.ca (M.M.); mariusz.klobukowski@ualberta.ca (M.K.); 2 Department of Biochemistry, University of Alberta, Edmonton, AB T6G 2H7, Canada; qw7@ualberta.ca (Q.W.); marek.michalak@ualberta.ca (M.M.); 3 Department of Physics, University of Alberta, Edmonton, AB T6G 2E1, Canada; 4 Dipartimento di Ingegneria Meccanica e Aerospaziale (DIMEAS), Politecnico di Torino, I-10129 Turin, Italy

**Keywords:** scoulerine, microtubule, cancer treatment, drug discovery, protein docking, molecular dynamic simulation

## Abstract

Scoulerine is a natural compound that is known to bind to tubulin and has anti-mitotic properties demonstrated in various cancer cells. Its molecular mode of action has not been precisely known. In this work, we perform computational prediction and experimental validation of the mode of action of scoulerine. Based on the existing data in the Protein Data Bank (PDB) and using homology modeling, we create human tubulin structures corresponding to both free tubulin dimers and tubulin in a microtubule. We then perform docking of the optimized structure of scoulerine and find the highest affinity binding sites located in both the free tubulin and in a microtubule. We conclude that binding in the vicinity of the colchicine binding site and near the laulimalide binding site are the most likely locations for scoulerine interacting with tubulin. Thermophoresis assays using scoulerine and tubulin in both free and polymerized form confirm these computational predictions. We conclude that scoulerine exhibits a unique property of a dual mode of action with both microtubule stabilization and tubulin polymerization inhibition, both of which have similar affinity values.

## 1. Introduction

**Figure 1:**
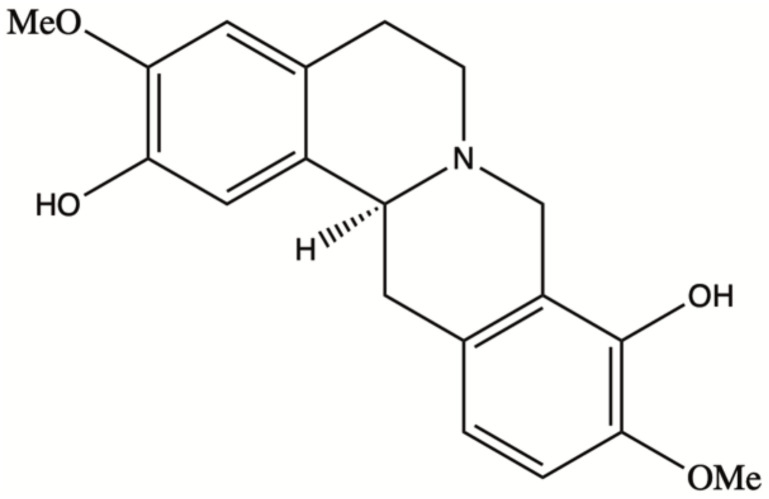
Scoulerine structure.

Natural products have played a dominant role in traditional medicine in the previous centuries. In recent years, in spite of major advances in the computational drug discovery and total synthesis areas, there has been a growing interest in using natural products for the development of anti-cancer therapeutics [1]. Some of these pharmaceutical agents have shown promising results in the prevention or treatment of cancer [2]. Scoulerine (also known as discretamine and aequaline) is a natural product isolated from Corydalis plants and belongs to one of the largest groups of natural compounds known as isoquinoline alkaloids [3]. Isoquinoline alkaloids are biogenetically derived from phenylalanine and tyrosine, having a basic structure of an isoquinoline or a tetrahydroisoquinoline ring in their scaffold [4]. Scoulerine molecule consists of two tetrahydroisoquinoline rings with two hydroxyls and two methoxyl functional groups (Figure 1). This molecule has shown a broad range of pharmacological properties such as antiemetic, antitussive, anti-bacterial, and anti-inflammatory activities [3]. It has also been demonstrated to have an anti-proliferative and pro-apoptotic function in cancer cells [5]. In addition, it is a precursor in the biosynthesis of noscapine, another natural compound with anti-mitotic properties that has been extensively tested in the cancer chemotherapy space [6,7,8,9].

Scoulerine inhibits β-site amyloid precursor protein cleaving enzyme 1 (BACE1), which is a very favourable target for Alzheimer’s treatment [10]. It has been also recently reported that scoulerine exhibits effective antimitotic activity, which leads to microtubule disruption, suggesting this molecule as a promising candidate for suppression of cancer cell growth [5].

Microtubules are ubiquitous filamentous structures found in the cytoskeleton of all eukaryotic cells. They polymerize from α/β tubulin heterodimers. Microtubules are dynamic polymers in kinetic equilibrium with α/β tubulin heterodimers in solution, which is achieved through polymerization and depolymerization cycles [11]. Microtubules play a crucial role in the development and maintenance of cell shape. They are also importantly involved in mitosis, motor transport, and cellular movements [12]. Microtubules have been one of the most commonly considered targets for tubulin-targeting chemotherapeutic agents. The α/β tubulin heterodimers and microtubules have several different binding domains. Some of the well-studied inhibitors and their binding pockets are: the colchicine-binding domain, vinca-binding domain, laulimalide-binding domain, and taxol-binding domain, to list only the most important few [11]. Most of the binding sites are not exclusive to primary inhibitors and can be targeted by other compounds. The mechanism of action of a large number of chemically diverse inhibitors of microtubules can be classified into two categories: they can act as either stabilizers or destabilizers. Microtubule-stabilizing agents stabilize the polymer by inhibiting depolymerization and inducing the polymerization of tubulin [13]. Microtubule-destabilizing agents bind to the tubulin dimers and destabilize microtubules by halting polymerization of tubulin [14]. Despite the known effects of scoulerine on microtubules, a precise mechanism of action of this molecule is still unclear and further research is required [5].

The present study aims to address the mode of action of scoulerine by means of computational prediction studies augmented by limited-scope experimental validation. For this purpose, blind docking was used to predict binding pockets for scoulerine. An evaluation scheme based on binding affinities and root mean square deviation (RMSD) between the crystallographic and the docked ligand conformations leads to valuable initial information. For an expanded investigation into predicted binding sites for scoulerine, molecular dynamics (MD) simulations were used. The complex systems of scoulerine bound in the potential binding pockets were designed and analyzed by RMSD and clustering analysis. All of the above-mentioned steps were followed to predict the stability of the binding interactions and closeness of the inhibitor to the potential scoulerine binding sites.

## 2. Result and Discussion

### 2.1. Scoulerine in Cancer Cell

To investigate the mechanism of scoulerine action it is essential to establish the proper structure for the ligand in the cancer cell environment. Scoulerine has a nitrogen atom in its ring that can be protonated in a sufficiently acidic environment. The acidity of cancer cells is slightly different from normal cells. In vivo, the extracellular matrix of tumours shows acidity ranging from 6.2 to 6.9 pH. However, the intracellular matrix of tumours is alkaline, having a pH range of 7.12 to 7.65 [15].

Structures and total energies of the five species involved in the scoulerine equilibria in aqueous environment, namely, scoulerine C_19_H_21_NO_4_ (Sco), scoulerine protonated at the nitrogen atom C_19_H_22_NO_4_^+^ (ScoH^+^), H_2_O, H_3_O^+^, and OH^−^

Sco_(aq)_ + H_3_O^+^_(aq)_ ⇄ ScoH^+^_(aq)_ + H_2_O _(aq)_, (1)

Sco_(aq)_ + H_2_O _(aq)_ ⇄ ScoH^+^_(aq)_ + OH^−^_(aq)_, (2)

were computed via quantum mechanical calculations using the Gaussian16 program [16]. The geometries of these species were optimized using the APFD density functional with dispersion [17] with the aqueous environment represented using the polarizable continuum model [18]. Two augmented basis sets of comparable quality were employed: Dunning’s correlation consistent basis aug-cc-pVDZ [19] and Pople’s 6−311++G (2d,p) basis [20]. The harmonic vibrational analysis confirmed that all stationary points were at their energy minima. The total electronic energies corrected for thermal free energies (i.e., the Gibbs free energies) and the (total) Gibbs free energy changes ΔG for the two equilibria are collected in Table 1.

The Gibbs free energy changes in the two equilibria show that while in an acidic environment, scoulerine will be protonated, and in the alkaline environment inside cancer cells, scoulerine will remain in its native form, with the equilibrium of Reaction (2) shifted to the left.

### 2.2. Analysis of Potential Scoulerine Binding Sites on β Tubulin

The AutoDock software package was used [21] to test whether it is possible to find the potential binding sites and binding modes of flexible scoulerine on α and β tubulin monomers without any prior knowledge of their location and conformation. The AutoDock-based blind docking (BD) approach [21] searches the entire surface of proteins for putative binding sites while simultaneously optimizing the conformations and the pose of the docked ligands. AutoDock is an appropriate tool for such a test because of its parameter set, based on the AMBER force field [22], and the capability of using flexible torsions for the ligands during the docking process. The protocol for docking procedures in different software packages is slightly different. In Autodock4, the auto-grid program maps the target protein first and then the AutoDock program docks the desired ligands to the set of grids of the mentioned protein [21].

**Figure 2:**
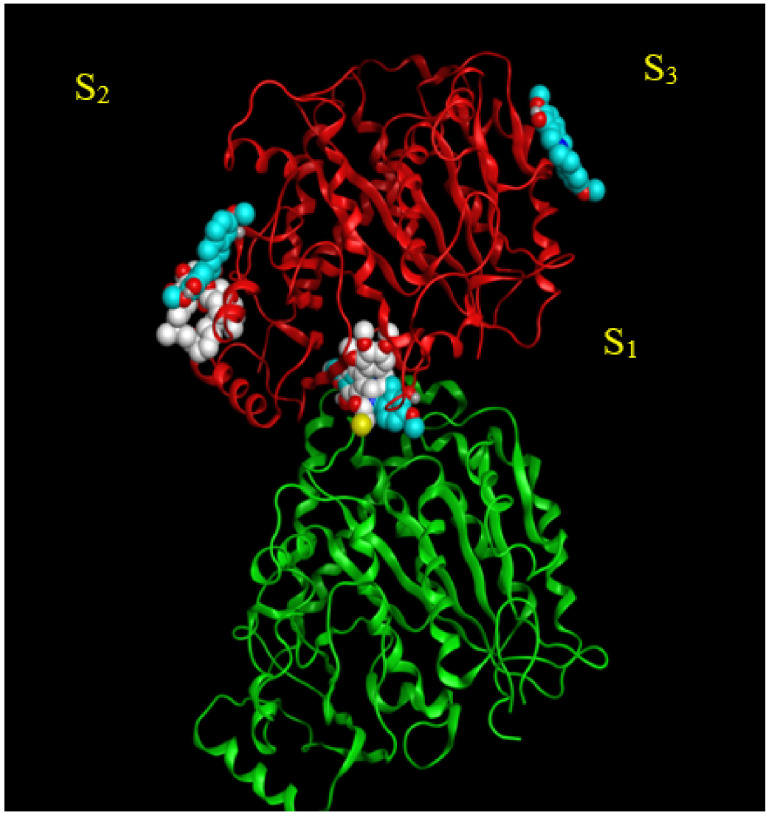
S_1_, S_2_, and S_3_ represent the three predicted potential binding sites by blind docking of scoulerine (blue) to α (green) and β (red) tubulins of 1SA0 PDB structure. Colchicine derivative from 1SA0 in S_1_ and laulimalide from 404H in S_2_ are shown in white.

Three potential binding sites were predicted by blind docking of deprotonated scoulerine to 1SA0 structure from the PDB (Protein Data Bank) (Figure 2). All three estimated binding sites were found to be located on the β tubulin monomer. To investigate whether any of the predicted binding sites matched with the known binding sites on β tubulin, 41 Protein Data Bank files were superimposed on the 1SA0 PDB structure with scoulerine docked to the three predicted binding sites. Vinca alkaloids, colchicine, taxol, epothilone, and laulimalide sites are the major binding sites for most stabilizing and destabilizing tubulin binding agents that bind to prevent the dynamics of microtubules [23].

CN2, a colchicine derivative, from the 1SA0 file and colchicine from the 5NM5 file, were found to be close to the docked scoulerine location in S_1_. This observation suggests that the S_1_ site has the potential to be a colchicine binding site. Laulimalide from the 4O4H file was also found to be close to the docked scoulerine location in S_2_. Based on the analysis, the S_2_ site can also potentially be a laulimalide binding pocket. However, regarding S_3_, none of the available inhibitors was close enough to the docked scoulerine.

### 2.3. Binding Affinities and Pose Analysis of Potential Scoulerine Binding Sites

To obtain numerical parameters to illustrate how close the potential binding sites are to the available colchicine and laulimalide binding sites, the root–mean–square deviation, RMSD, values of scoulerine in S_1_ and S_2_ were calculated with respect to the reference crystal structures of colchicine, CN2 (a colchicine derivative) and laulimalide form the 5NM5, 1SA0, and 4O4H PDB files, respectively.

To calculate the RMSD of scoulerine to the reference crystal structures, colchicine was docked to the colchicine binding site in the 5NM5 and 1SA0 structures. The crystal complex was aligned and superimposed on the docked complex to confirm that the chosen method of docking can predict the correct pose and conformation of colchicine. Then scoulerine was docked to the colchicine binding site in the same position with the same approach. The α/β heterodimer tubulin structures with the docked scoulerine to the colchicine binding site of the 1SA0 structure were aligned and superimposed with the crystal structure, 1SA0 PDB. The adjacent atoms between the two superimposed ligands were paired manually between the backbone of the ligands. The RMSD was calculated based on the distance between the paired atoms by MGLtool 1.5.7 (The Center for Computational Structural Biology (CCSB), La Jolla, CA, USA).

The RMSD values of 3.5 and 3.4 Å between blind-docked scoulerine in S_1_ and the crystal structure of colchicine (5MN5) and CN2 (1SA0) support the assumption and illustrate that the colchicine might share its binding site with scoulerine. The same method was applied to laulimalide and scoulerine with respect to the binding site of laulimalide based on the 4O4H PDB structure. The RMSD values of 1.6 Å display even more adjacency between the docked scoulerine in S_2_ and the crystal structure of laulimalide (4O4H) (Table 2).

To put to a test the strength of interactions between scoulerine and the residues of the colchicine binding site, colchicine and scoulerine were docked specifically to the colchicine binding site (1SA0) by AutoDock and their binding affinities were then compared (Table 3). The same method was applied to calculate and compare the binding affinities of laulimalide and scoulerine to the only crystal structure that is available for the laulimalide binding site (4O4H). The fact that laulimalide is docked between adjacent microtubule protofilaments and perhaps has two binding sites on β tubulin should not be overlooked (Table 3).

The binding affinity of −9.23 kcal/mol for colchicine versus −7.96 kcal/mol for scoulerine in the same binding site of β tubulin predicts weaker interactions between scoulerine and the colchicine binding site of β tubulin. Scoulerine is a new chemotherapeutic compound and most of the biological aspect of the drug still needs to be evaluated. In 2018, the Habartova group used 20 μM of scoulerine to disrupt microtubule function in the A549 lung cancer cell line where nocodazole, another colchicine binding site inhibitor (CBSI), was used as a control [6]. Nocodazole, at a concentration of 5 µM was shown to be as effective as scoulerine [5,24]. A binding affinity of −7.5 kcal/mol for laulimalide versus −6.87 kcal/mol for scoulerine in the same binding site of β tubulin also indicates weaker binding interactions between scoulerine and β tubulin in the laulimalide binding site of the 4O4H PDB crystal structure (Table 3).

The steps described below were followed to evaluate the three potential binding sites on β tubulin and identify which one might be the most probable binding site for scoulerine. First, visualization of the docked poses of scoulerine was performed. Next, an analysis of the interacting residues of each binding site on β tubulin with scoulerine was carried out. Finally, results of molecular dynamics simulations of scoulerine in colchicine and laulimalide binding pockets were inspected.

### 2.4. Colchicine Site

**Figure 3:**
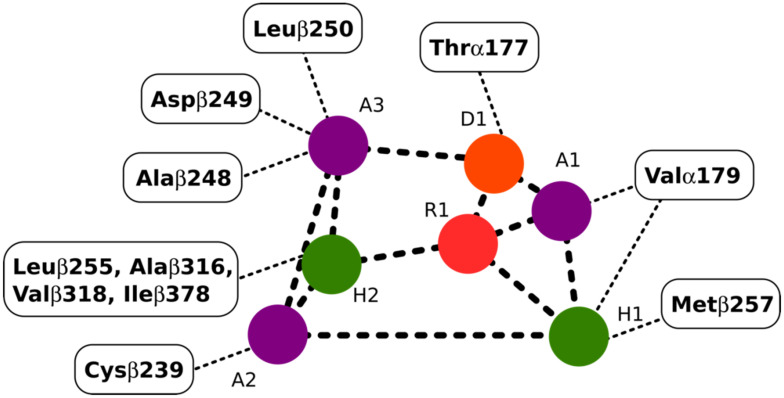
Interactions between the pharmacophoric points and the tubulin structure. Seven pharmacophoric points: three hydrogen bond acceptors (A1, A2, and A3) in purple dots, one hydrogen bond donor (D1) in an orange dot, two hydrophobic centers (H1 and H2) in green dots, and one planar group (R1) in a red dot. Figure 3 was designed based on the information provided in the source [25].

The colchicine binding site on tubulin is a well-studied binding pocket and to date, many crystal structures of inhibitors have been found to dock in the colchicine binding site [25,26]. Seven pharmacophoric points were distinguished for CBSIs and are displayed in Figure 3. Based on previous work done on the subject, none of the known structures of CBSIs contains all seven pharmacophore groups [25,26]. Three hydrogen bond acceptors of pharmacophoric points are labelled as A1, A2, and A3 in Figure 3. The backbone nitrogen of Valα179 of the colchicine binding pocket is in contact with A1. The sulfur atom of Cysβ239 interacts with A2. Finally, A3 forms one contact mainly with the backbone nitrogen of Alaβ248, Aspβ249, and Leuβ250. The hydrogen bond donor of pharmacophoric points, D1, interacts with the backbone oxygen of Thrα177. H1 and H2 are the two hydrophobic centers of pharmacophoric points. The H1 point reacts to the side chains of Valα179 and Metβ257. H2 interacts with side chains of Leuβ255, Alaβ316, Valβ318, and Ileβ378. The last pharmacophoric points, R1, belong to one planar group (Figure 3) [25,26].

#### 2.4.1. Potential Scoulerine Binding Site (S_1_)

**Figure 4:**
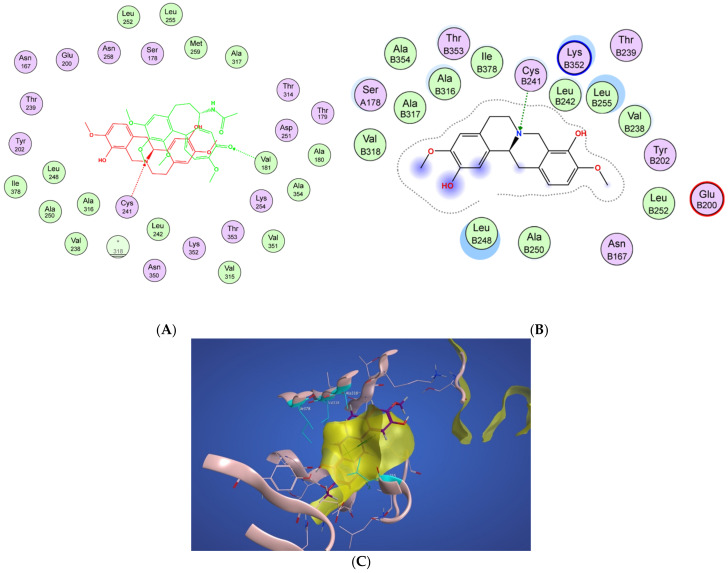
(**A**) Two-dimensional interaction scheme of the superimposed colchicine crystal structure from the 5NM5 PDB file (green) on scoulerine in the S_1_ site (red) on 1SA0. Star (*) on residue 318 indicates two different amino acids on 5NM5 and 1SA0 structures. (**B**) Two-dimensional interaction scheme of scoulerine in the S_1_ site. (**C**) Surface patches identifying regions of hydrophobicity (yellow) around scoulerine. Residues Leu255, Ala316, Val318, and Ile378 of β tubulin that are involved in hydrophobic interactions are colored in teal.

In Figure 4A, a two-dimensional interaction scheme of the superimposed colchicine crystal structure from the 5NM5 PDB file (green) on scoulerine in the S_1_ site (red) illustrates the binding pose of scoulerine in comparison to the binding pose of colchicine. Even though the pose of the colchicine crystal structure overlaps with the pose of scoulerine in S_1_ (Figure 4A), analyzing the adaptation of scoulerine with seven pharmacophore groups of colchicine binding site inhibitors was essential. The two-dimensional interaction scheme (Figure 4B) displays interactions between scoulerine and a potential binding pocket, S_1_. Scoulerine has the A1 pharmacophoric point of CBSI ligands because of the hydrogen acceptor interaction between a sulfur atom of Cys239 with N of scoulerine. The A3 pharmacophoric point of CBSI ligands is supposed to have a hydrogen acceptor by the backbone nitrogen of Ala248 or Leu250. However, the distance between the backbone nitrogen of Ala248 or Leu250 and scoulerine is 4.2 Å which translates into weak electrostatic interactions. Taking into consideration that the pose of scoulerine is the result of blind docking, there is a possibility that a small adjustment might lead to hydrogen bonding with either Ala248 or Leu250 (see Figure 4B). The third pharmacophoric point of CBSI, H2, is a hydrophobic center that interacts with side chains of Leu255, Ala316, Val318, and Ile378. The green color of the above-mentioned residues in the 2-dimensional interaction scheme in Figure 4B means a greasy property, which refers to the hydrophobic nature of the residues. The blue circles show the ligand exposure to the solvent and the dotted line around the ligand shows the proximity contour. The closer the ligand is to the contour in the scheme, the deeper the ligand’s placement in the cavity of the binding pocket of the protein. To illustrate it more clearly, Figure 4C shows the hydrophobic surface of the protein in the S_1_ site that wraps around the hydrophobic center, H_2_, of scoulerine.

Scoulerine also has a planar group that corresponds to the pharmacophoric R1 point. The D1 and A1 pharmacophoric points of CBSI interact with Thr177 and Val178 of α tubulin. However, the closest residue of α tubulin in Figure 4B is Ser178.

#### 2.4.2. Conformational Analysis

##### RMSD and RMSF Analysis on S_1_ Site

**Figure 5:**
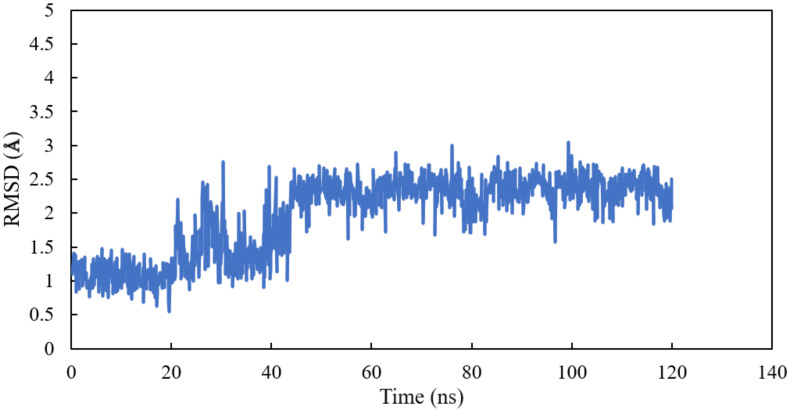
RMSD of scoulerine in the colchicine binding site.

Homology modelling of human α and βI tubulin based on the 1SA0 template was performed. Scoulerine was specifically docked to the colchicine binding site. A molecular dynamics simulation of the system was performed for 120 ns. All atoms except hydrogens of α and β tubulin monomers were fitted to their structure after equilibration and the RMSD values of scoulerine were calculated while the backbone of the colchicine binding site was fitted during the simulation. To assess the equilibration of the system, the plot of total energies of the system versus time was generated and compared to the RMSD plot. The system appeared to be equilibrated after 43 ns of simulation time. The RMSD value in the range from 2.2 to 2.3 Å for a 77 ns simulation after the equilibration verified that the interactions between scoulerine and residues of the colchicine binding site are strong enough to keep the ligand close to the binding pocket (Figure 5).

**Figure 6:**
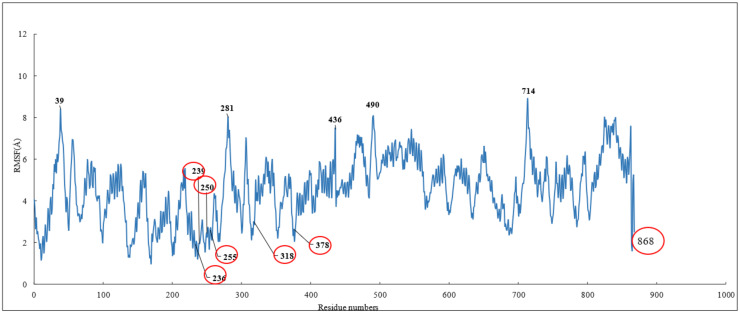
RMSF of all residues and scoulerine in the colchicine binding site.

To investigate the stability of the pose of scoulerine in the colchicine binding site of α and β tubulin, the root–mean–square fluctuation (RMSF) of all protein residues and ligands was calculated during 120 ns of simulation (Figure 6). The residues in the binding site, namely 236, 239, 250, 255, 318, and 378, that contribute to the binding interaction with scoulerine show small values of RMSF in the range between 1.0 and 2.5 Å. Scoulerine, labelled as 868, also shows a small RMSF value, 2.5 Å, that confirms the interaction with binding residues is strong enough to not let the ligand fluctuate substantially. The residues 39, 281, 436, 490, and 714 show the largest range of RMSF, from 7.5 to 8.5 Å. These residues are far from any of the scoulerine, GTP, and GDP ligands in the structure.

**Figure 7:**
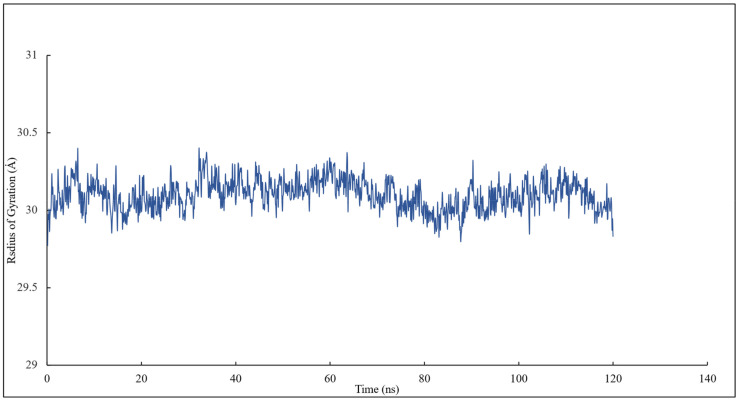
The radius of gyration of all residues and scoulerine in the colchicine binding site.

The radius of gyration of all the residues of α and β tubulins and scoulerine in the colchicine binding site was calculated to study the compactness of target-ligand complexes over a 120 ns simulation. The value of the radius of gyration fluctuates between 29 and 30 Å. The result indicates that the compactness of the system is stable during 120 ns of the simulation (Figure 7).

##### Clustering Analysis

Clustering analysis was carried out with the hierarchical agglomerative algorithm [27]. Several studies have discussed and validated the use of hierarchical algorithms in MD simulations [28,29]. The frames of 77 ns were clustered as reported by binding site closeness. To be specific, this closeness was sorted based on the mass-weighted RMSD of the binding-site atoms, which includes scoulerine and residues having atoms within 8 Å of scoulerine. The centroid structures have the smallest RMSD relative to all the other members of the same cluster.

**Figure 8:**
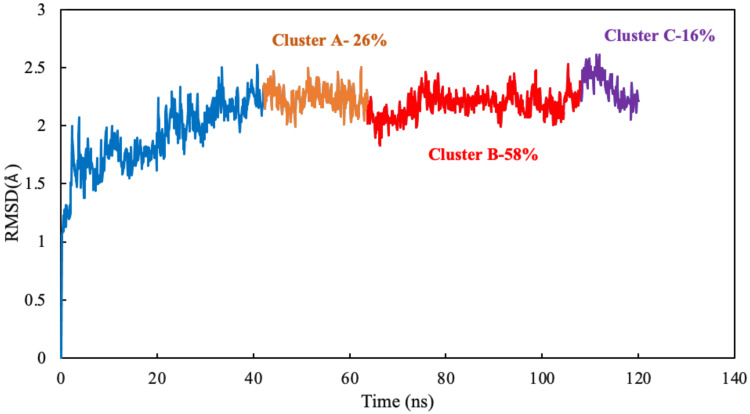
Mass-weighted root–mean–squared deviation (Å) of the binding site of colchicine on tubulin, classified according to the cluster number, with occupancy indicated. The binding site includes scoulerine and residues having atoms within 8 Å of scoulerine. The dark blue part of the graph illustrates the equilibration phase of the simulation.

**Figure 9:**
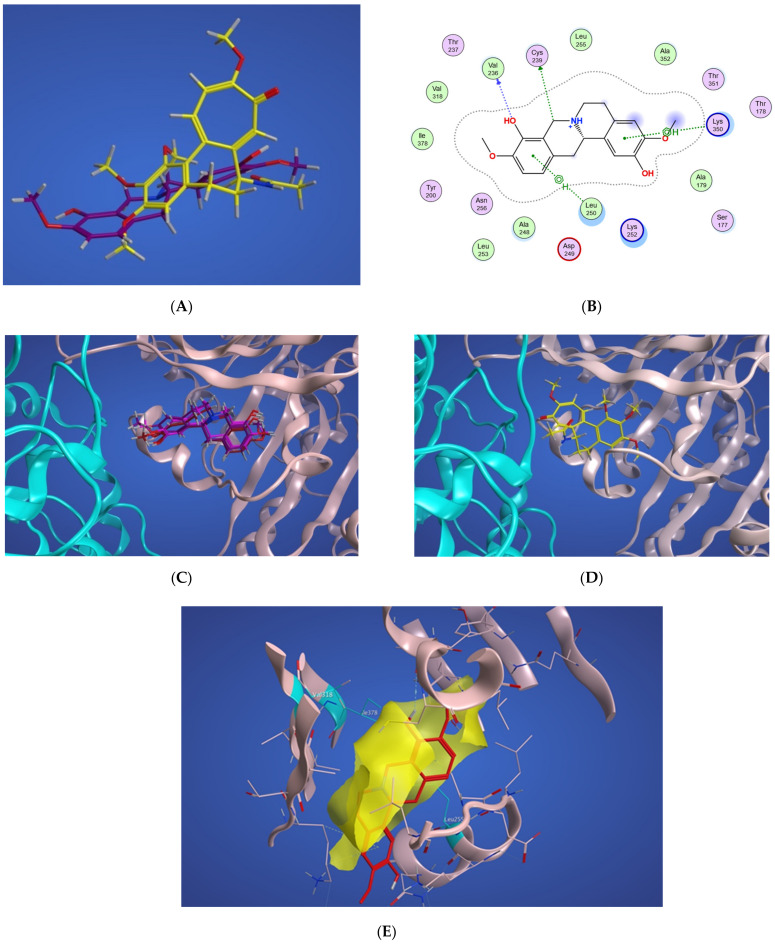
(**A**) Representative structures of scoulerine in cluster B (purple) versus colchicine (yellow). (**B**) Two-dimensional interaction scheme of scoulerine in the colchicine binding site. (**C**) Representative structures of cluster A (red), cluster B (purple), and cluster C (dark pink) in the colchicine binding site, α tubulin colored in teal, and βI tubulin colored in light pink. (**D**) Colchicine (yellow) in the colchicine binding site, α tubulin colored in teal, and βI tubulin colored in light pink. (**E**) Surface patches identifying regions of hydrophobicity (yellow) around scoulerine, residues Leu255, Val318, and Ile378 of β tubulin that are involved in hydrophobic interaction colored in teal.

The algorithm generates representative structures, centroid structures, of scoulerine poses in the colchicine binding site throughout the 77 ns simulation. The trajectory frames were partitioned into clusters A, B, and C (Figure 8). Cluster B of the graph indicates more than 50 percent of occupancy during the simulation. In Figure 9A, the pose of the representative structure of dominant cluster B was displayed with the pose of colchicine’s crystal structure (Figure 9D) of the 5NM5 structure. The representative structure (centroid) for each cluster was extracted and displayed in Figure 9C.

As displayed in Figure 9C, the sulfur atom of Cysβ239 still has a hydrogen acceptor with scoulerine (A2). As predicted before, the backbone nitrogen of Leuβ250 now is sufficiently close to produce hydrogen binding with scoulerine (A3). Hydrophobic interactions between scoulerine (H2) with side chains of Leu255, Val318, and Ile378 still occurred as illustrated in Figure 9E. As mentioned above, in the interaction diagram of blindly docked scoulerine to α/β tubulin, the interaction of D1, a pharmacophoric point of colchicine binding site inhibitors, with the backbone oxygen of Thrα177 is not fulfilled. However, the interaction diagram of the most dominant representative structure of scoulerine docked to the colchicine binding site of human α/βI tubulin heterodimer over 77 ns of MD simulation shows Thrα177 being near enough to the ligand to demonstrate a weak electrostatic interaction. 

### 2.5. Laulimalide Binding Sites on β Tubulin

**Figure 10:**
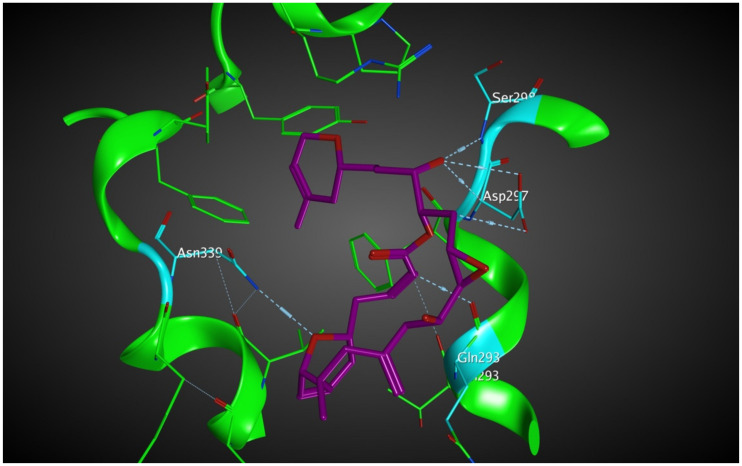
Laulimalide in the laulimalide binding site of β tubulin (green) based on the 4O4H PDB file. The residues in blue are involved hydrogen bond interaction with laulimalide (purple).

Laulimalide is a relatively novel microtubule stabilizer that binds between two protofilaments of a microtubule, which has been in the spotlight because of its unique mode of action. Following computational studies that attempted to identify the laulimalide binding site, the first crystal structure of laulimalide bound to tubulin was captured by x-ray diffraction in 2014. The binding pocket is formed by residues Gln293, Phe296, Pro307, Arg308, Tyr312, Val335, Asn339, Tyr342, Ser298, Asp297, and Phe343 of tubulin (Figure 10). Gln293, Ser298, Asp297, and Asn339 are the residues that make hydrogen bonds with laulimalide [27,30,31].

Computational studies investigating the mode of action of laulimalide discovered the Gln293, Phe296, and Asn 339 residues of β tubulin as the most stabilizing residues [28,31,32]. These computational analyses also showed that the Lys122, Glu125, Ser126, and Arg121 residues of β tubulin in the adjacent protofilament bind to laulimalide but they have smaller stabilizing contributions [27,30,31].

Similar to the colchicine binding pocket, laulimalide is not the only inhibitor that binds to the laulimalide binding sites. Peloruside (4O4L PDB) is another drug candidate that binds to the laulimalide binding site of β tubulin as has been identified by X-ray diffraction. The binding mode of peloruside and laulimalide to tubulin is homogeneous. In this case, Ser298, Asp297, Arg308, Gln293, and Tyr312 residues of tubulin form hydrogen bonds with peloruside. Gln293, Ser298, and Asp297 residues are special since they make hydrogen bonds with both inhibitors, laulimalide and peloruside [31].

#### 2.5.1. Potential Scoulerine Binding Site (S_2_)

**Figure 11:**
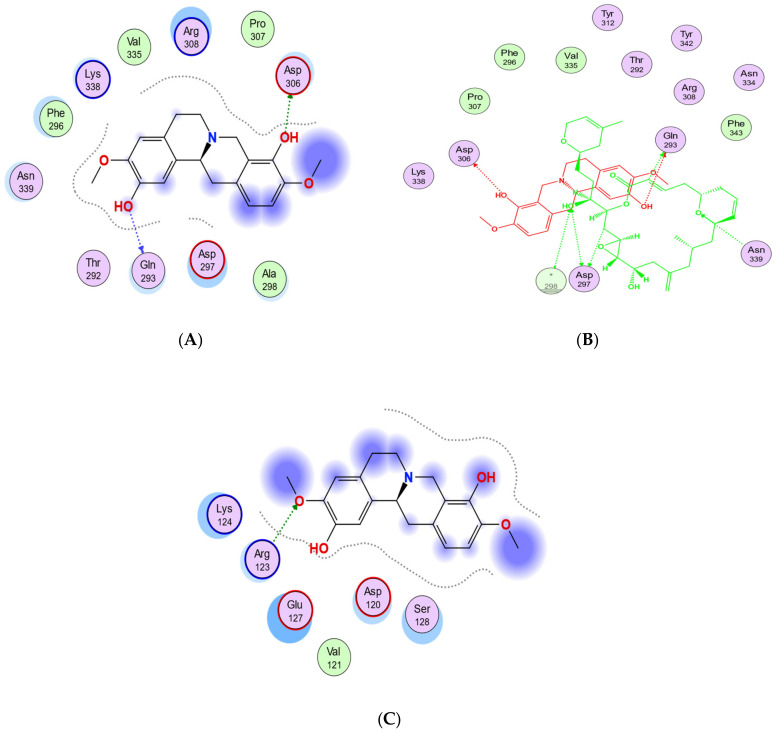
(**A**) Two-dimensional interaction scheme of scoulerine in the S_2_ site identified by blind docking. (**B**) Two-dimensional interaction scheme of the superimposed laulimalide crystal structure based on the 4O4H PDB file (green) with scoulerine (red) in the S_2_ site on 1SA0. Star (*) on residue 298 indicates two different amino acids on 4O4H and 1SA0 structures. (**C**) Two-dimensional interaction scheme of scoulerine in the S_3_ site found via blind docking.

Based on the blind docking results, the O37 of the hydroxyl group of scoulerine in the binding site S_2_, similarly to laulimalide and peloruside, makes hydrogen-donor bonds with the side chains of Gln293 (Figure 11A). Asp297 of the laulimalide binding pocket also forms hydrogen bonds with laulimalide and peloruside. However, in the interaction of scoulerine with the residues of the S_2_ site, Asp297 shows electrostatic interaction instead. Pro307, Arg308, Val335, Lys338, Phe296, and Asn339 are other interactive residues in the S_2_ site that are in common with the residues of the laulimalide binding site. In Figure 11B, a two-dimensional interaction scheme of the superimposed laulimalide crystal structure from the 4O4H PDB file (green) with scoulerine in the S_2_ site (red) illustrates the pose of scoulerine in comparison with the pose of laulimalide.

These computational analyses also showed that the Lys122, Glu125, Ser126, and Arg121 residues of β tubulin in the adjacent protofilament bind to laulimalide but they have a smaller stabilizing contribution.

The S_3_ site is primarily identified by blind docking of scoulerine to the 1SA0 PDB structure and does not show any compatibility with available binding sites of β tubulin revealed by crystallography (Figure 11C). The residues of the S_3_ site, namely Arg123, Lys124, Glu127, and Ser128, are very similar to the residues of the second binding site of laulimalide on β tubulin in the adjacent microtubule protofilament, namely Lys122, Glu125, Ser126, and Arg121.

#### 2.5.2. Conformational Analysis

RMSD analysis was performed on scoulerine bound between adjacent microtubule protofilaments (laulimalide binding sites). Homology modeling of the human α/βI tubulin heterodimer was based on the 4O4H crystal structure combined with the 2XRP crystal structure to arrange two adjacent protofilaments. The scoulerine pose was taken from scoulerine docked in the laulimalide binding site on 4O4H [31].

**Figure 12:**
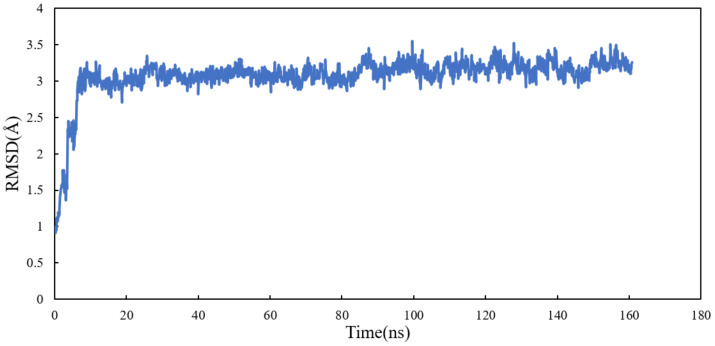
RMSD plot of scoulerine docked to the laulimalide binding sites.

Molecular dynamics simulation of the system was performed for 160 ns. All atoms except hydrogens of α and β tubulin monomers were fitted to their structure after equilibration and the RMSD values of scoulerine were calculated while the backbone of the laulimalide binding sites were fitted during the simulation. To assess the system’s equilibration, the plot of total energies of the system versus time was generated and compared to the RMSD plot. The system appeared to be equilibrated after 10 ns but since substantial structural equilibration (45 ns) is necessary to stabilize the lateral contacts between neighbouring tubulin heterodimers, production data were collected for 115 ns after equilibration. The RMSD values ranging from 3.1 to 3.3 Å for 115 ns of simulation verified that the interactions between scoulerine and the residues in the scoulerine binding site are strong enough to keep the ligand close to the binding pocket (Figure 12).

**Figure 13:**
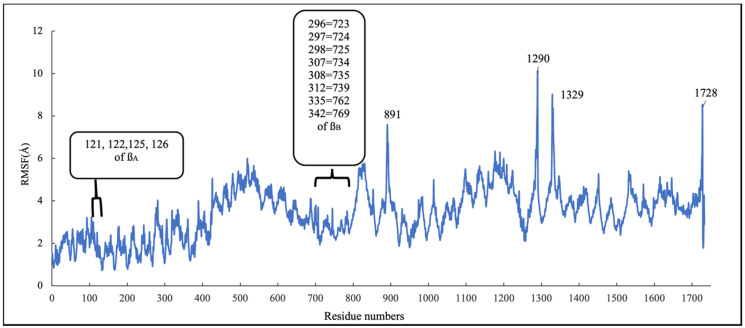
RMSF of all residues and scoulerine in laulimalide binding sites.

To investigate the stability of the pose of scoulerine in the laulimalide binding sites of α_A_ and β_A_ and α_B_ and β_B_ tubulins, the root–mean–square fluctuation (RMSF) values for all protein residues and ligands were calculated during 160 ns of the simulation (Figure 13). The residues in the laulimalide site on β_A_ tubulin, 121, 122, 125, and 126, that contribute to the binding interaction with scoulerine, show small values of RMSF in the range between 1.0 and 2.0 Å. The residues 296, 297, 298, 307, 308, 312, 335, and 342 of the laulimalide site on β_B_ tubulin also show a moderate range of RMSF values from 2.5 to 3.5 Å. Scoulerine, identified as 1729, also shows a small RMSF value, 2.5 Å, which confirms the interaction with binding residues is strong enough to not let the ligand fluctuate. The above-mentioned results imply that the interaction of scoulerine is stronger with the laulimalide binding site on β_A_ tubulin in comparison to the adjacent laulimalide binding site on β_B_ tubulin.

The residues 891, 1290, 1329, and 1728 show the largest range of RMSF, from 7.7 to 9.8 Å. Note that these residues are far from any of the scoulerine, GTP, and GDP ligands in the structure.

**Figure 14:**
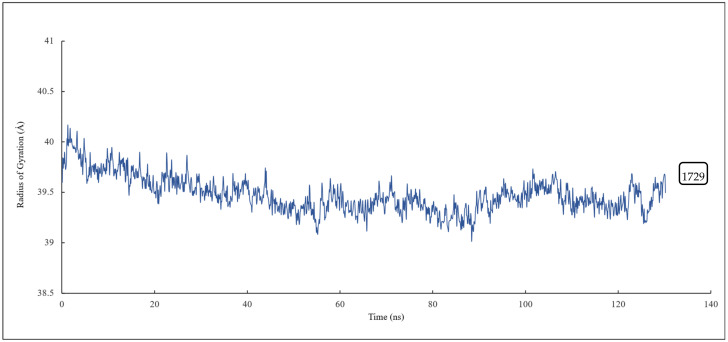
The radius of gyration of all residues and scoulerine in the laulimalide binding sites.

The radius of gyration of all the residues of α and β tubulin monomers and scoulerine in the laulimalide binding sites of α_A_ and β_A_ and α_B_ and β_B_ tubulins were calculated to study the compactness of the target–ligand complexes following 120 ns of simulation. The value of the radius of gyration fluctuates around 39 Å. The result indicates that the compactness of the system is stable during 120 ns of the simulation (Figure 14).

##### Clustering Analysis

**Figure 15:**
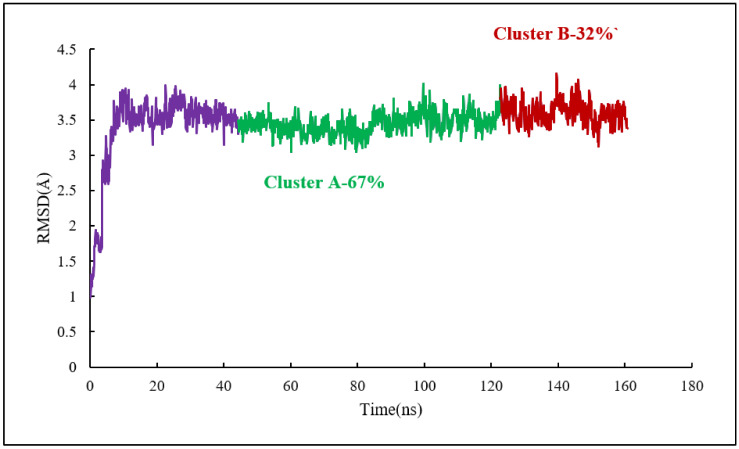
Mass-weighted root–mean–squared deviation (Å) of the binding sites of laulimalide to tubulin, classified according to cluster number, with their occupancy indicated. The binding site includes scoulerine and tubulin residues whose atoms are within 8 Å of scoulerine. The purple part of the graph illustrates the equilibration phase of the simulation.

As for the colchicine binding site, clustering analysis was also conducted for the frames of the last 115 ns of the simulation to show the stability of the protein system as it keeps the ligand in the binding pockets. The mass-weighted RMSD of the binding-site atoms throughout the trajectory frames of 115 ns were classified after equilibration to two clusters. To be specific, the binding-site atoms include scoulerine and residues having atoms within an 8 Å radius of scoulerine, with water and ions being excluded. The algorithm also generates two representative structures of scoulerine poses in the laulimalide binding sites between the two adjacent protofilaments for each of the clusters (Figure 15). Cluster A of the graph indicates more than 67 percent of occupancy during the simulation.

**Figure 16:**
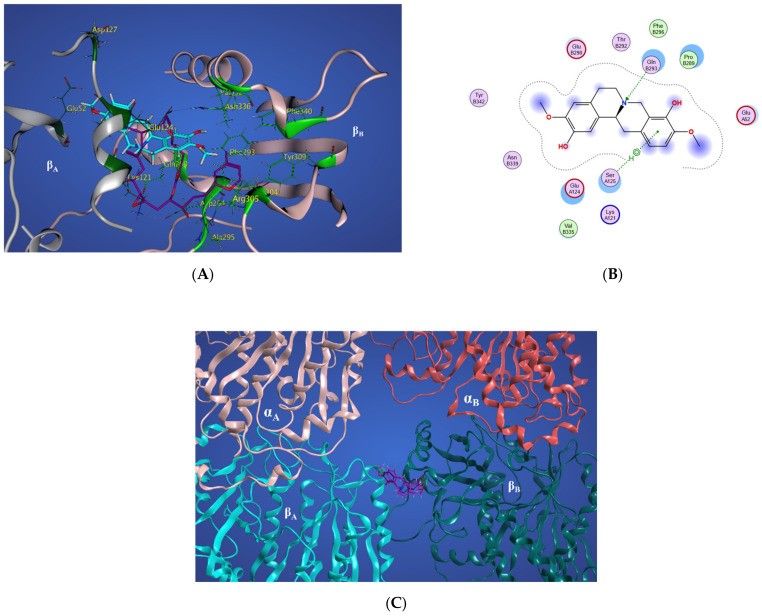
(**A**) Three-dimensional interaction scheme of scoulerine (blue) and a superimposed laulimalide crystal structure from the 4O4H PDB file (purple) between microtubule protofilaments. Residues shown in light green are in the laulimalide site on β_A_ tubulin and residues shown in dark green are in the laulimalide site on β_B_ tubulin. (**B**) Two-dimensional interaction scheme of scoulerine in the laulimalide binding sites on β_A_ tubulin and β_B_ tubulin. (**C**) Representative structures of cluster A (purple) and cluster B (pink) in the laulimalide binding sites. α_A_ and α_B_ tubulins colored in light and dark pink and β_A_ and β_B_ tubulins colored in light and dark green, respectively.

Representative structures of scoulerine between α_A_ β_A_ and α_B_ β_B_ tubulin monomers of two adjacent protofilaments are displayed in Figure 16C. Representative structures for cluster A are shown in purple and in dark pink for cluster B, respectively.

In Figure 16A, the representative structure of the dominant cluster A is displayed with the superimposed laulimalide crystal structure from the 4O4H PDB file. The residues of the laulimalide binding pocket of β_B_ tubulin are highlighted in light green. The computational study illustrated the residues of the second binding site of laulimalide on the adjacent β_A_ tubulin and they are colored as dark green in Figure 16A [27]. The 2D interaction scheme of the most dominant representative structure of the system shows that scoulerine can also bind between β tubulins of two adjacent microtubule protofilaments (Figure 16B). The hydrogen acceptor between the nitrogen of scoulerine and Gln293 of β tubulin and the π-hydrogen interaction between a ring of scoulerine and Ser125 of β_A_ tubulin, are the two most important binding interactions between scoulerine and the residues of laulimalide binding pockets. Gln293, Phe296, and Asn339 residues of β tubulin are the most important stabilizer residues for the binding interaction between laulimalide and the residues of its tubulin binding sites. The involvement of all three residues in the interaction scheme of scoulerine with the laulimalide binding sites [27,30] raises the possibility that scoulerine might be a new type of inhibitor that can bind between two microtubule protofilaments. Additionally, Val335 and Phe296 residues of the laulimalide binding site also showed weak electrostatic interaction with scoulerine. As shown in Figure 16A, scoulerine has a smaller-size structure compared to laulimalide. Thus, this new compound may shift its binding location from the first binding pocket for laulimalide on β_B_ tubulin, based on the crystal structure of the laulimalide binding site 4O4H PDB, toward the second one on β_A_ tubulin to be able to bind to both binding sites. Note that Lys122, Glu125, and Ser126 are the most important residues in the laulimalide binding pocket on β_A_ tubulin [27,30], which also interact with scoulerine (Figure 16A,B).

### 2.6. Experimental Validation

Based on the computational predictions presented above, scoulerine can potentially bind to both the colchicine and laulimalide binding sites. However, based on docking results, the binding affinities might not be as strong as either colchicine or laulimalide.

To experimentally estimate the dissociation constant of scoulerine bound to free α and β tubulin dimers and microtubules, respectively, assays involving the microscale thermophoresis method were performed in this study. Rhodamine labelled free α and β tubulin dimers and microtubules were used for the thermophoresis experiments. The experimental setup consists of an infrared laser coupled into the path of fluorescent excitation/emission. The laser is focused onto the sample through the same objective that is used for fluorescence detection, which allows the monitoring of thermophoresis in a microfluidic sample compartment such as capillaries.

**Figure 17:**
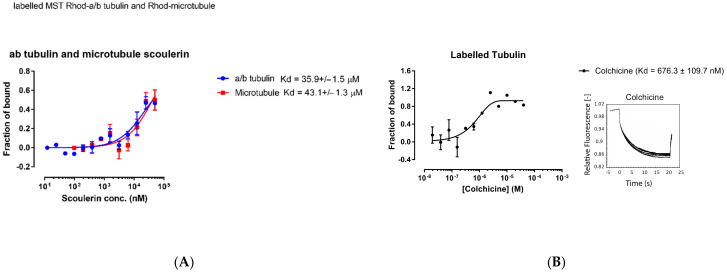
(**A**) Purified porcine αβ tubulin (colchicine binding site, blue circle) and microtubule (laulimalide binding site, red square) binding to scoulerine via microscale thermophoresis. Each data point represents the mean of two independent measurements and the error bar is shown as the standard deviation. The binding curve is fitted with Graphpad Prism 7.0. (**B**) Fluorescence labelled purified porcine α/β tubulin binding to colchicine. Normalized microscale thermophoresis time traces are shown on the right. Each data point is the mean of three independent microscale thermophoresis measurements; error bars represent the standard deviation. The binding curve is fitted with Graphpad Prism 7.0.

Inside the thermophoresis capillaries, the aqueous solution is heated locally via infrared laser at the center, creating a spatial temperature gradient. Molecules (rhodamine labelled free α and β tubulin dimers or microtubules with titrated scoulerine) will start moving along the temperature gradient and reach equilibrium concentration distribution at steady state. To measure thermophoresis of proteins, the change in concentration between the initial state and the steady state is measured. The movement of the molecules is affected by the surface area, effective charge, and the hydration entropy of the molecule-solution interface. The binding of scoulerine to rhodamine labelled free α and β tubulin dimers or microtubules will change one of these parameters, resulting in different thermophoresis of the complex comparing the lowest scoulerine concentration solution to the highest scoulerine concentration solution. The binding kinetics K_d_ can be calculated with a series of scoulerine titrations. The K_d_ values of 35.9 × 10^−6^ M and 43.1 × 10^−6^ M were reported for scoulerine bound to labelled free α/β tubulin heterodimers and labelled microtubules, respectively (Figure 17A).

The range of values for the reported dissociation constants supports the computational results and indicates that scoulerine can bind to both free tubulin dimers and microtubules. Consequently, we conclude that scoulerine has a dual mechanism of action.

For quantitative comparison, the dissociation constant, K_d_, of the well-studied colchicine bound to free α/β tubulin heterodimers was also measured using the same method to provide a reference value. The measured K_d_ value of 6.76 × 10^−7^ M shows that colchicine’s binding affinity is stronger than that of the scoulerine in the interaction with tubulin dimers (Figure 17B). 

The binding affinities calculated via docking were reported to be −9.32, −7.96, and −6.87 kcal/mol for colchicine and scoulerine in the colchicine and laulimalide binding sites, respectively (Table 3). The corresponding K_d_ values of 1.89 × 10^−7^, 1.64 × 10^−6^, and 9.9 × 10^−6^ were calculated based on the computed binding affinities for colchicine, scoulerine in the colchicine and laulimalide binding sites, respectively. The theoretical K_d_ value of 1.89 × 10^−7^ vs. the experimental value of 6.76 × 10^−7^ shows that the docking approach overestimates the strength of binding interactions between colchicine and α and β tubulin. The theoretical value of 9.9 × 10^−6^ vs. the experimental value of 43 × 10^−6^ for scoulerine in the laulimalide binding sites and 1.64 × 10^−6^ vs. 35 × 10^−6^ for scoulerine in the colchicine binding sites confirm the same problem. This is not surprising since computational predictions of binding energies commonly require recalibration based on experimental benchmarks. Docking software is designed to virtually screen large libraries of compounds in a short period of computational time and works well for rank ordering these compounds but not so well for obtaining precise binding energy values. The method used in our study did involve more accurate but also more time-consuming algorithms such as the molecular mechanics Poisson–Boltzmann (MM/PBSA) or Generalized Born (MM/GBSA) surface area continuum solvation methods, which are superior to docking at predicting binding free energies. However, our predicted values are still accurate enough to correctly compare the relative affinities of the compared compounds based on the strength of their estimated binding energies. Unfortunately, due to extreme difficulty in obtaining samples of laulimalide, we have not been able to test its binding affinity for tubulin or microtubules in this assay. However, results of such assays have been reported elsewhere [32]. The range of values of binding affinities is consistent with the reported dissociation constant values.

## 3. Materials and Methods

### 3.1. 3D Structure Preparation of the Ligand

The two-dimensional (2D) chemical structure of scoulerine C_19_H_21_NO_4_ (Sco), scoulerine protonated at the nitrogen atom C_19_H_22_NO_4_^+^ (ScoH^+^), H_2_O, H_3_O^+^, and OH^−^ were converted into a corresponding three-dimensional (3D) structure. Total energies of the five species were computed via quantum mechanical calculations using the Gaussian16 program (Gaussian, Inc., Wallingford, USA) [16]. The geometries of these species were optimized using the APFD density functional with dispersion [17] with the aqueous environment represented using the polarizable continuum model [18]. Two augmented basis sets of comparable quality were employed: Dunning’s correlation consistent basis aug-cc-pVDZ [19] and Pople’s 6–11++G (2d,p) basis [19,20]. The harmonic vibrational analysis confirmed that all stationary points were at their energy minima. The total electronic energies corrected for thermal free energies (i.e., the Gibbs free energies) and the (total) Gibbs free energy changes.

### 3.2. Blind Docking

The optimized structure of scoulerine was blindly docked to the 1SA0 Protein Data Bank (PDB) structure of the α/β tubulin heterodimer via AutoDock4 software [21]. To do so, the maximum size of the grid box used was 126 × 126 × 126 Å^3^, which then divided each tubulin monomer into three parts and a docking procedure was subsequently applied. The grid center coordinates of the grid box for scoulerine in the colchicine binding site are x = 114.728, y = 87.376, and Z = 7.774 (all in Å). For the second box, the grid center of the grid box for scoulerine in the laulimalide binding site of β_B_ tubulin is: x = 8.565, y = 15.72, and z =−7.006 (all in Å). The grid center given by x = 2.374., y = 17.316, and Z = 0.21. Then, 136 Å was applied for the last box, i.e., scoulerine in the laulimalide binding site of β_A_.

### 3.3. 3D Structure Preparation of Complexes for MD Simulation

#### 3.3.1. Scoulerine in the Colchicine Binding Site

The complex designed in the first part of the present study consists of scoulerine bound in the colchicine-binding pocket of human α/βI tubulin heterodimers. A homology model allows us to overcome the obstacle of not having a valid crystal structure for human α (TBA1A_HUMAN) and βI tubulin monomer (TBB5_HUMAN). The software package MOE2018 (Molecular Operating Environment, Inc) [33] was used to perform the homology modelling procedure. The 1SA0 PDB crystal structure [34] was used as a structural template to create human α/βI tubulin heterodimers based on the corresponding sequence (UniProt: P07437) for human βI and (UniProt: Q71U36) for human α tubulin. The scoulerine structure was optimized by quantum mechanics/molecular mechanics (QM/MM) calculations. The pose of the drug was taken from the docked scoulerine to the colchicine binding site of the 1SA0 PDB crystal structure.

#### 3.3.2. Scoulerine in the Laulimalide Binding Sites of Microtubule

The model used in the second part of the present study consists of scoulerine bound between two adjacent tubulin heterodimers. The homology models of the human βI tubulin (TBB5_HUMAN) sequence (UniProt: P07437) and human α tubulin (TBA1A_HUMAN) sequence (UniProt: Q71U36) were generated by taking tubulin structures in 4O4H as a template [31]. The protofilament arrangement was based on the 2XRP crystal structure, which combined 8 Å resolution cryo-electron microscopy data with the 4O4H crystal structure, which has a resolution of 2.1 Å, in order to obtain a microtubule structure at an atomic resolution [31,35]. The scoulerine pose was taken from the docked scoulerine to the laulimalide binding site in 4O4H.

#### 3.3.3. Molecular Dynamic Simulations

In both complexes, parameters for scoulerine were compatible with the general Amber force field (GAFF) and calculated via the antechamber suite of Amber 18 [36]. The Amber ff12SB force field was used to describe tubulin components. Each complex was solvated in an octahedral box of TIP3P water molecules [37] extending 12 Å from the solute. To obtain a 0.15 M ion concentration, sodium and chloride ions were added to neutralize the systems. The systems were gradually heated up to 310 K over 200 ps and maintained at 310 K for another 100 ps under constant volume conditions (NVT). The Langevin thermostat was used with a time collision frequency of 2 ps. Non-bonded terms were calculated within a 10 Å cut-off, except for long-range electrostatics, which was calculated with the particle mesh Ewald method [38]. During simulations, the SHAKE algorithm was used [39].

#### 3.3.4. Clustering Analysis

RMSD-based clustering was used to extract protein and ligand structures to represent the overall closeness and stability of a new inhibitor in the binding site. The movement trajectory of the complex was broken down into clusters of similar sampled conformations during the MD simulation. The mass-weighted RMSD of the tubulin components, fitted to the heavy atoms of the backbone of the protein, was calculated with respect to the structure at 0 ns. The clustering analysis was performed on each system, which was structurally equilibrated after 43 ns using one of the bottom-up algorithms, the average-linkage, in AmberTools18 (Figure 6 and Figure 12) [40]. Several studies have discussed and validated the use of hierarchical algorithms in MD simulations [28,29]. A representative structure, a centroid structure, was extracted for each cluster and used for comparative analyses [41].

#### 3.3.5. Microscale Thermophoresis

Microscale thermophoresis analyses were carried out using a Monolith NT.115 instrument (Nano Temper Technologies, München, Germany). For tubulin binding to colchicine, lyophilized tubulin powder was purchased from commercial sources (Cytoskeleton Inc., Denver, CO, USA; T240) and reconstituted as previously described (Kalra et al., 2020). Briefly, 180 µL of GTP (guanosine triphosphate) supplemented BRB80 (80 mM PIPES pH 6.9, 2 mM MgCl_2_, 0.5 mM EGTA, 1mM GTP) was first added to 20 µL of microtubule cushion buffer (BRB80T in 60% glycerol). This solution was added to 1 g of lyophilized tubulin powder for reconstitution, aliquoted and stored at −80 °C. Reconstituted tubulin was labeled with RED-NHS fluorescence labelling kit (Nano Temper Technologies, cat# MO-L001) following the manufacturer’s protocol. Experiments were carried out at 23 °C in Monolith NT.115 Premium capillaries (Nano Temper Technologies, cat# MO-L011), with 40% LED power (fluorescence lamp intensity) and 60% microscale thermophoresis power (IR-laser intensity). The assay buffer contained 80 mM PIPES-KOH, pH 6.9, 2 mM MgCl_2_, and 0.5 mM EGTA, with a final DMSO concentration of 0.4% for colchicine. Three replicates for colchicine binding to labelled tubulin were performed.

For tubulin and microtubule binding to scoulerine, rhodamine labelled tubulin (Cytoskeleton Inc., Denver, CO, USA; TL590m; 20 µg) was reconstituted by adding 70 µL of unlabeled tubulin solution (described above) to 5 µL of microtubule cushion buffer. All experiments were carried out at 23 °C in Monolith NT.115 Premium capillaries (Nano Temper Technologies, cat# MO-L011), with 95% LED power (fluorescence lamp intensity) and 60% microscale thermophoresis power (IR-laser intensity). Scoulerine was diluted into the assay buffer containing 80 mM PIPES-KOH, pH 6.9, 2 mM MgCl_2_, and 0.5 mM EGTA, with a titration range of 50 µM to 12.2 nM. Experiments were performed in two replicates at 22 °C. All data were analyzed by Monolith Affinity Analysis v2.2.6 software, exported to excel and plotted with GraphPad Prism 7.0 (Nano Temper Technologies, München, Germany).

## 4. Conclusions

Scoulerine is a natural compound, which is a member of the family of isoquinoline alkaloids that can be extracted from *Croton flavens* [42], *Corydalis dubia* [43], and *Corydalis cava* [10,44]. Recent research on scoulerine has revealed a range of effects, including anti-proliferative and pro-apoptotic properties, as well as antimitotic activity that disrupts microtubules [5,6]. The listed properties of scoulerine make it a possible candidate for use in cancer treatment. However, the mode of action of scoulerine is still unclear to date. The present work attempted to predict the mechanism of action of this new chemotherapeutic agent using a computational approach augmented by simple experimental validation assays. A combination of blind docking and molecular dynamics provides a useful approach to acquiring new, detailed information about the interactions between scoulerine and β tubulin both as a free unit and within a microtubule. Three potential binding sites were found on β tubulin of a microtubule via the blind docking method. With the help of RMSD between the crystallographic structure of inhibitors of β tubulin and the docked ligand conformations, three possible binding sites have been discovered and labelled S_1_, S_2_, and S_3_ (Figure 1). The residues of the discovered S_1_ binding site on β tubulin are mostly the same as the colchicine binding pocket.

Laulimalide is a unique stabilizer of the microtubule that can bind to β tubulins of adjacent protofilament [27]. The residues comprising the predicted S_2_ and S_3_ binding sites on β tubulins have similarities with the laulimalide binding site on β tubulins belonging to adjacent protofilaments. Two improved models of scoulerine binding to α/β tubulin heterodimers were designed and investigated by molecular dynamics simulations. The first one consists of scoulerine located between α and β tubulins in the crystallographic colchicine binding sites based on the 1SA0 PDB file. In the second one, scoulerine is placed between two adjacent α/β heterodimers and bound to a crystallographic laulimalide binding site based on 4O4H PDB. The cluster analyses were performed for both of the systems. The structure and 3D interaction scheme of the representative structure of the highest cluster for both systems were also displayed. The results showed that scoulerine can bind between both α and β tubulin monomers within a single heterodimer. It can also bind between β tubulins of two adjacent heterodimers. This computational prediction was put to a test by measuring the dissociation constant between scoulerine bound to labelled free tubulin dimers and labelled microtubules. The K_d_ values of 35.9 × 10^−6^ M and 43.1 × 10^−6^M were reported for scoulerine bound to labelled free α/β tubulin heterodimers and labelled microtubules, respectively. The similarity between the values of the K_d_ for both systems is consistent with the computational estimations and indicates that scoulerine might have a dual mechanism of action both as a microtubule stabilizer in the laulimalide binding sites and an inhibitor of microtubule polymerization, which binds in the colchicine binding site. This places scoulerine in a unique category of tubulin-binding agents.

## Figures and Tables

**Table 1:** T The total electronic energies, corrected for thermal free energies (in the Hartree atomic units, E_h_; 1 E_h_ = 627.51 kcal/mol) and the (total) Gibbs free energy changes ΔG for the two equilibria (in kcal/mol).

Level of Theory	Sco	ScoH^+^	H_2_O	H_3_O^+^	OH^−^	ΔG_(1)_	ΔG_(2)_
APFD/aug-cc-pVDZ	−1091.278	−1091.716	−76.385	−76.764	−75.885	−37.0	+38.9
APFD/6-311++(2d,p)	−1091.443	−1091.882	−76.400	−76.779	−75.899	−37.7	+38.9

**Table 2:** RMSD values for scoulerine in S_1_ and S_2_ with respect to the reference of crystal structures of colchicine, colchicine derivative, CN2, and laulimalide form 5NM5, 1SA0, and 4O4H PDB files, respectively.

Crystal Structure (Reference)	Docked Scoulerine	RMSD (Å)
1SA0 (CN2)	S_1_	3.4
5NM5 (colchicine)	S_1_	3.5
4O4H (laulimalide)	S_2_	1.6

**Table 3:** A—Binding energies of scoulerine and colchicine docked in the colchicine binding site (1SA0). B—scoulerine and laulimalide docked in the laulimalide binding site (4O4H).

	Colchicine Binding Site A		Laulimalide Binding Site B	
Name	colchicine	scoulerine	Laulimalide	scoulerine
B.A (kcal/mol)	−9.23	−7.96	−7.50	−6.87

## Data Availability

All data that generated or analysed during this study are available from the corresponding authors upon justified request.

## Sample Availability

Samples of the compounds can be obtained from the authors by request.
